# Delayed TBI-Induced Neuronal Death in the Ipsilateral Hippocampus and Behavioral Deficits in Rats: Influence of Corticosterone-Dependent Survivorship Bias?

**DOI:** 10.3390/ijms24054542

**Published:** 2023-02-25

**Authors:** Ilia Komoltsev, Daria Shalneva, Olga Kostyunina, Aleksandra Volkova, Stepan Frankevich, Natalia Shirobokova, Anastasia Belikova, Sofia Balan, Olesya Chizhova, Olga Salyp, Daria Bashkatova, Pavel Kostrukov, Aleksandra Soloveva, Margarita Novikova, Natalia Gulyaeva

**Affiliations:** 1 Department of Functional Biochemistry of the Nervous System, Institute of Higher Nervous Activity and Neurophysiology, Russian Academy of Sciences, Moscow 117485, Russia; 2 Moscow Research and Clinical Center for Neuropsychiatry, Moscow 115419, Russia

**Keywords:** traumatic brain injury, hippocampus, corticosterone, memory, neurodegeneration, neuroinflammation, microglia, survivorship bias

## Abstract

Acute and chronic corticosterone (CS) elevations after traumatic brain injury (TBI) may be involved in distant hippocampal damage and the development of late posttraumatic behavioral pathology. CS-dependent behavioral and morphological changes were studied 3 months after TBI induced by lateral fluid percussion in 51 male Sprague–Dawley rats. CS was measured in the background 3 and 7 days and 1, 2 and 3 months after TBI. Tests including open field, elevated plus maze, object location, new object recognition tests (NORT) and Barnes maze with reversal learning were used to assess behavioral changes in acute and late TBI periods. The elevation of CS on day 3 after TBI was accompanied by early CS-dependent objective memory impairments detected in NORT. Blood CS levels > 860 nmol/L predicted delayed mortality with an accuracy of 0.947. Ipsilateral neuronal loss in the hippocampal dentate gyrus, microgliosis in the contralateral dentate gyrus and bilateral thinning of hippocampal cell layers as well as delayed spatial memory deficits in the Barnes maze were revealed 3 months after TBI. Because only animals with moderate but not severe posttraumatic CS elevation survived, we suggest that moderate late posttraumatic morphological and behavioral deficits may be at least partially masked by CS-dependent survivorship bias.

## 1. Introduction

The pathogenesis of traumatic brain injury (TBI) is believed to be mediated by neuroinflammation and neurodegeneration [1]. In the hippocampus, an area selectively vulnerable to excitotoxic and ischemic impacts, glucocorticoid receptors are highly expressed and regulate stress responses [2]. Acute and chronic corticosterone (CS) elevation may be an important systemic mechanism underlying the selective death of hippocampal neurons and neuroinflammation after TBI. Hippocampal atrophy was shown in patients after TBI [3], and it was associated with posttraumatic epilepsy [4] and anxiety symptoms [5]. Lateral fluid percussion brain injury (LFPI) in rats is the most commonly used TBI model [6]. LFPI induces early astrogliosis in the neocortex [7,8,9] and also causes distant hippocampal damage [10,11,12]. Transient impairments of performance in fear conditioning [13], learning and memory deficits in the Morris water maze [6,14] after LFPI were also reported. In a previous study, we showed CS elevation on day 3 after LFPI and reported that neuronal cell loss was detected bilaterally 7 days after lateral fluid percussion brain injury in rats (LFPI), starting in the ipsilateral hippocampus earlier than in the contralateral one [15,16]. We also showed the time-dependent effects of CS associated with bilateral neuroinflammation during the acute posttraumatic period.

Glucocorticoid-dependent morphological and behavioral changes in the hippocampus may be closely associated with late posttraumatic pathology development [17]. In this study, we aimed to evaluate CS-dependent behavioral and morphological changes 3 months after TBI. It could be assumed that higher acute CS elevation and/or prolonged moderate CS elevation in blood could be associated with early and late cognitive posttraumatic disturbances in rats as well as with severe hippocampal damage 3 months after TBI.

## 2. Results

### 2.1. Time Course of Corticosterone during 3 Months after TBI and Mortality

**Figure 1:**
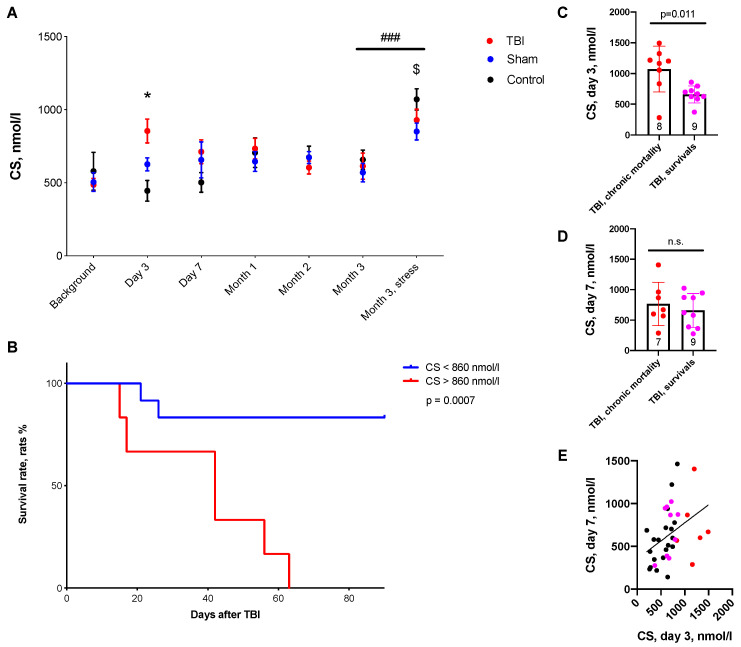
CS time course 3 months after TBI and TBI-induced mortality. (**A**)—CS dynamics during the experiment. CS was elevated on day 3 in rats after TBI. After a forced swim test (month 3, stress) CS was elevated in all animals, though it tended to be higher in controls. (**B**)—Survival rates in rats with high and low CS levels on day 3 after TBI. Survival rate was lower in rats with CS > 860 nmol/L (Kaplan–Meier method). (**C**,**D**)—CS levels on days 3 and 7 in survived animals and deceased animals, respectively (Wilcoxon test). (**E**)—Correlation of CS levels on day 3 and 7 (Spearman correlation, *p* = 0.007). *—*p* = 0.007, day 3 after craniotomy, Kruskal–Wallis test, TBI vs. Control, *p* = 0.011. $—*p* = 0.090, Kruskal–Wallis test, Sham vs. Control, *p* = 0.098. ###—*p* < 0.001, Wilcoxon test, n.s.—not significant.

Blood CS levels increased in the TBI group on day 3 after the injury (*p* = 0.007). No other differences between TBI, Sham and control groups could be detected. Three months after craniotomy, CS levels increased in all rats 30 min after the forced swim test and tended to be higher in controls (*p* = 0.09), reflecting changes in stress reactivity in animals after craniotomy (Figure 1A).

Elevated CS levels on day 3 predicted remote mortality (Figure 1B–E). Mortality in the acute period of TBI was 27% (7 rats), and cumulative mortality by the end of the second month was 58% (15 rats). Animals that died during the first month after TBI had higher blood CS levels on day 3 after the injury compared to TBI survivors (*p* = 0.011). The accuracy of mortality prediction by CS level on day 3 was assessed using ROC analysis (Figure 1 and Figure S1, Table S1). The accuracy of prediction for the cut-off CS value 860 nmol/L was 0.947. The survival rate was significantly lower in rats with CS > 860 nmol/L (*p* = 0.0007).

### 2.2. Behavioral Changes in Acute and Remote Periods of TBI

**Figure 2:**
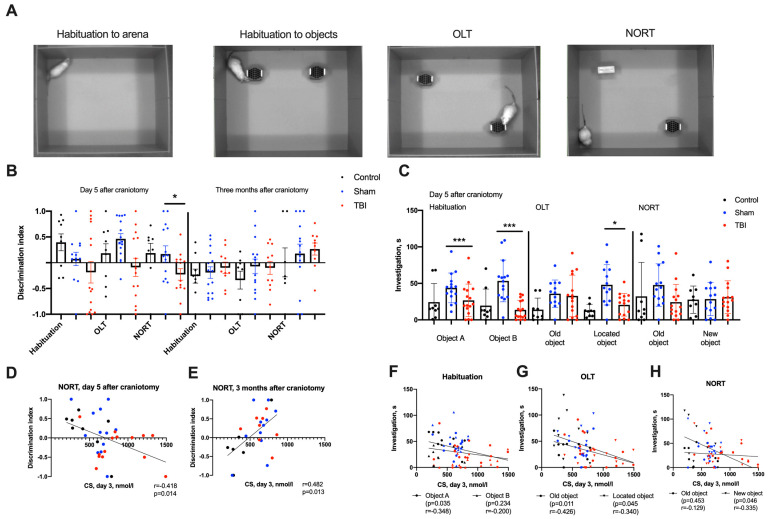
Behavior in OLT and NORT in acute and remote (3 months) periods after TBI. (**A**)—The order of the behavioral tests. (**B**)—Discrimination indices in OLT and NORT on day 5 and at 3 months after craniotomy. The discrimination index was lower in NORT during the acute posttraumatic period. (**C**)—Cumulative duration of object investigation in OLT and NORT during the acute posttraumatic period. Rats after TBI investigated objects less during both habituation and during OLT. (**D**,**E**)—Correlation of CS levels on day 3 after TBI with the discrimination index in NORT in the acute and remote posttraumatic periods (Spearman correlations). (**F**–**H**)—Correlation of CS levels on day 3 after TBI with cumulative durations of object investigation during habituation, OLT and NORT (Spearman correlations). *—*p* < 0.05, ***—*p* < 0.001, Kruskal–Wallis test with post-hoc Dwass–Steel–Critchlow–Fligner test.

Using new object recognition tests (NORT) in the acute period of TBI, we revealed differences in the object discrimination index: it was lower in the TBI group compared to the Sham and control groups (*p* < 0.05). The discrimination index correlated with acute CS elevation, indicating early corticosterone-dependent objective memory disturbance (Figure 2). However, CS elevations on day 3 positively correlated with the discrimination index in NORT 3 months after craniotomy in survivors, which is possibly due to survivorship bias. In object location tests (OLT), the discrimination index did not correlate with CS levels (Figure S2). 

Detailed analysis of animal behavior in OLT and NORT showed additional differences between groups. During the habituation session, the cumulative durations of object investigation and the latency of the first investigation were lower for both objects in the TBI group (*p* < 0.05), whereas the differences in distances moved and velocities showed a statistical trend only. In OLT, we found a reliable decrease in the cumulative investigation time of the moved object in the TBI group (*p* < 0.005).

The cumulative duration of investigation in all sessions correlated with CS elevation on day 3 after TBI: animals with higher CS levels investigated objects to a lesser extent during the habituation session, and the same was true for located and new objects (*p* < 0.05, Figure 2). Three months after TBI, no differences between groups could be found in either OLT or NORT.

**Figure 3:**
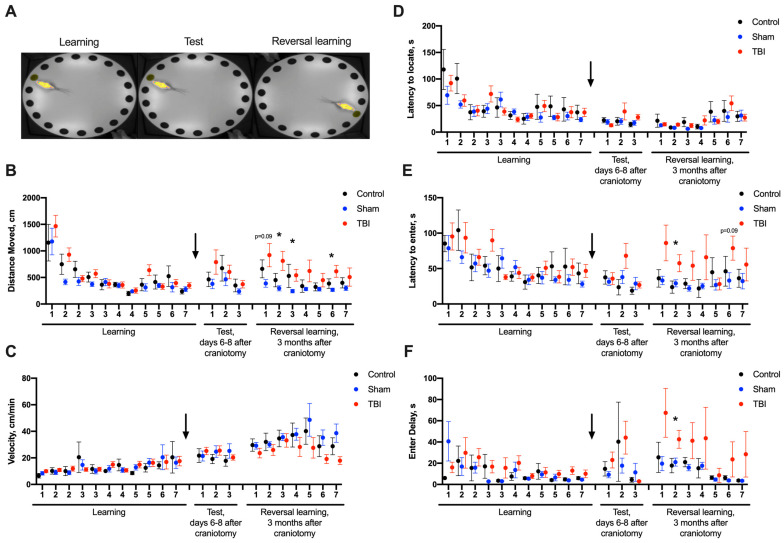
Behavior of rats in the Barnes maze in the acute and remote (3 months) periods after TBI. (**A**)—Order of behavioral assessments. (**B**)—Distance moved. Three months after craniotomy, animals after TBI tended to travel a longer distance to the previously learned position on test day 1. During reversal learning tests, the moved distances were higher on days 2, 3 and 6 of reversal learning 3 months after TBI. (**C**)—Velocity during tests. (**D**)—Latency to locate escape tunnel. (**E**)—Latency to enter escape tunnel. The latency to enter was higher in animals with TBI on days 2 and 6 of reversal learning 3 months after TBI. (**F**)—Enter delay. The delay to enter the escape tunnel before the first finding was higher on day 2 of reversal learning 3 months after TBI. *—*p* < 0.05, Kruskal–Wallis test. Black arrow indicates LFPI on the timeline.

In the Barnes maze, no differences between groups were found in the acute period of TBI. Three months after craniotomy, we observed a tendency to increase the distance moved to the escape tunnel from its initial position in the TBI group (*p* = 0.095). On day 2 (reversal learning), rats after TBI showed increases in distance traveled and time taken before entering the escape tunnel. On days 3 and 6, the distance moved was also increased in rats after TBI (Figure 3). On day 6 of reversal learning, a decreased proportion of rats with TBI directly targeted the escape tunnel (Fischer exact test, *p* = 0.086, Figures S3 and S4). These results demonstrated mild cognitive impairments during reversal learning 3 months after TBI. We found no significant correlations between CS levels and performance in the Barnes maze during the acute or chronic TBI periods.

In the Elevated Plus Maze (EPM) test performed during the acute period of TBI, rats from the TBI group spent more time in the open arms as compared to the Sham and control groups (*p* < 0.05). The number of rats looking out from the dark arms decreased, and the number of transitions between the light arm and the center increased (*p* < 0.05) in the TBI group. These changes did not correlate with CS levels (Figure S5). Three months after craniotomy in all experimental groups, both the speed and distance in the EPM test decreased compared to the test performed 3 months earlier. All animals preferred the dark compartment of the EPM (*p* < 0.001). In the open field test, no statistically significant differences between the TBI, Sham and control groups could be found in the acute or remote periods after trauma.

### 2.3. Neurodegeneration and Neuroinflammation in the Hippocampus 3 Months after TBI

**Figure 4:**
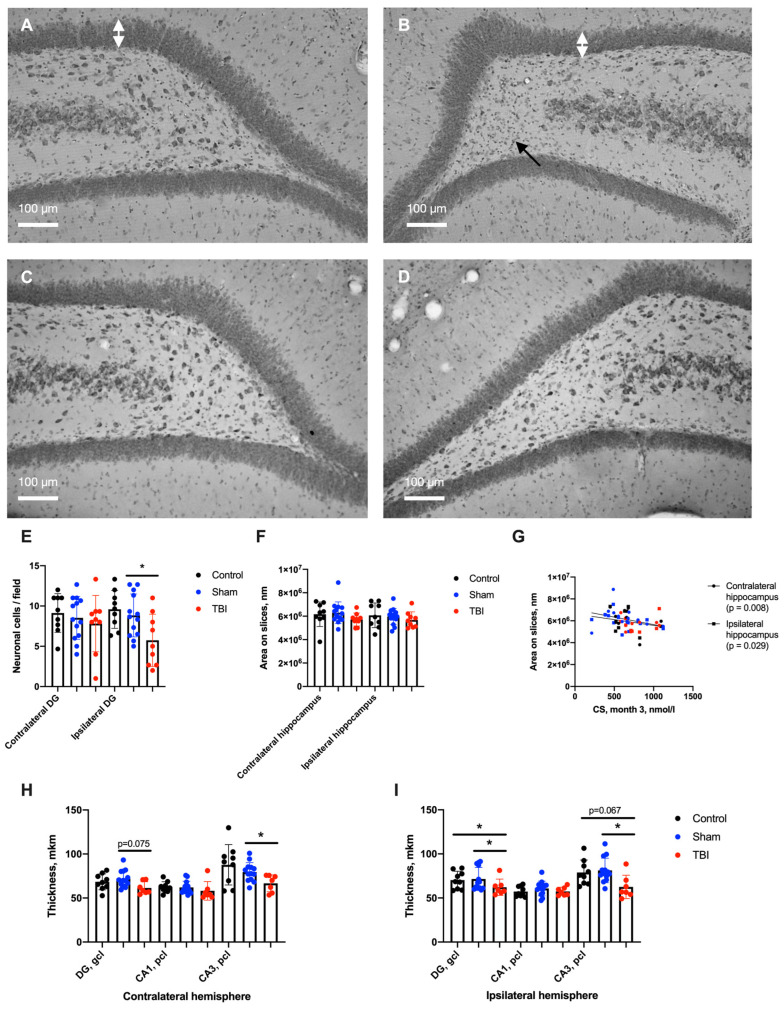
Neurodegeneration in the hippocampus 3 months after TBI. (**A**,**B**)—Contralateral and ipsilateral hippocampal DGs of rats after TBI, respectively; Nissl staining. White arrows show granular cell layer (gcl) thicknesses, and black arrows show areas of neuronal cell loss in the polymorph layer. (**C**,**D**)—Contralateral and ipsilateral hippocampal DGs of Sham rats, respectively; Nissl staining. (**E**)—Neuronal cell density in the hippocampus. The number of neurons was lower in the ipsilateral DG. (**F**)—Hippocampal area on slices. (**G**)—Correlation between hippocampal areas on slices and CS levels 3 months after TBI (Spearman correlation). (**H**,**I**)—Thickness of hippocampal cell layers in contralateral and ipsilateral hemispheres, respectively. Thinning of cell layers was observed in the granular cells layer (gcl) and pyramidal cells layer (pcl) in the CA3 of both hemispheres. *—*p* < 0.05, Kruskal–Wallis test with post-hoc Dwass–Steel–Critchlow–Fligner test.

Three months after TBI neuronal cell loss in the ipsilateral dentate gyrus (DG), the polymorph layer and the bilateral thinning of the hippocampal granular (*p* < 0.05 for ipsilateral and *p* = 0.075 for contralateral hemispheres) and pyramidal cell layers in the CA3 fields of both hemispheres (*p* < 0.05) were revealed (Figure 4). No changes in the whole hippocampal area on slices were detected, though we found a negative correlation of ipsilateral and contralateral hippocampal areas on slices with increased CS levels 3 months after TBI.

**Figure 5:**
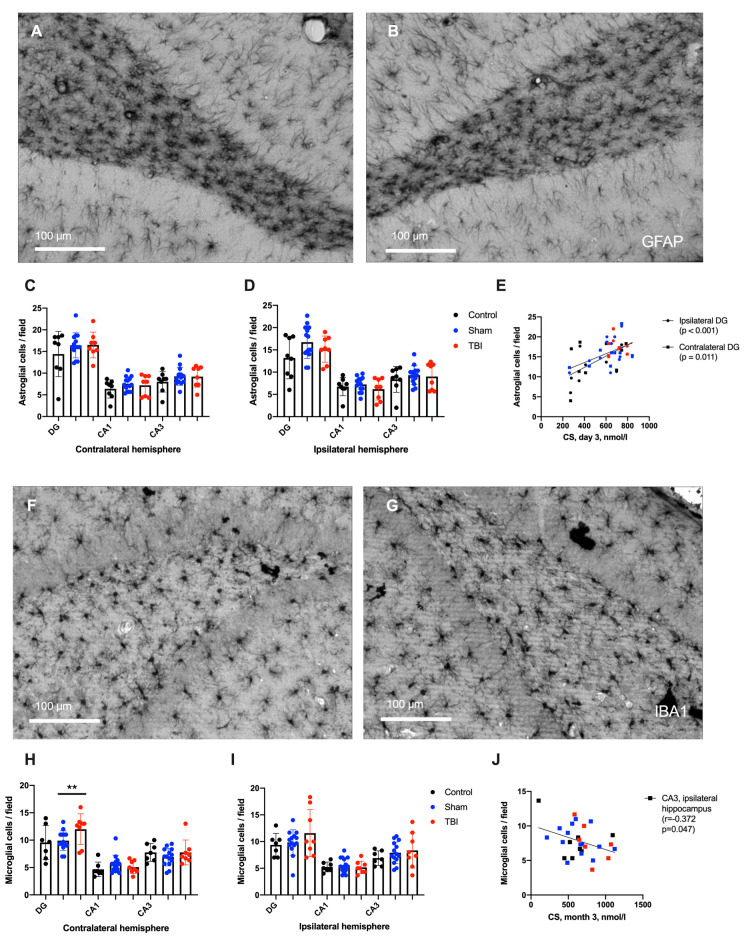
Gliosis in the hippocampus 3 months after TBI. (**A**,**B**)—Astroglial cells in contralateral and ipsilateral hippocampal DGs, respectively; anti-GFAP staining. (**C**,**D**)—Astroglial cell density in the contralateral and ipsilateral hemispheres, respectively. (**E**)—Correlation between astroglial cell density and acute CS levels on day 3 after TBI (Spearman correlation). (**F**,**G**)—Microglial cells in the contralateral and ipsilateral hippocampal DGs, respectively; anti-IBA1 staining. (**H**,**I**)—Microglial cell density in the contralateral and ipsilateral hemispheres, respectively. Microglial activation was detected only in contralateral DGs. (**J**)—Negative correlation between microglial cell density in CA3 and CS level 3 months after TBI (Spearman correlation). **—*p* < 0.01, Kruskal–Wallis test with post-hoc Dwass–Steel–Critchlow–Fligner test.

Changes in glial cell numbers were found only in the contralateral hemisphere; the number of microglial cells in the DG was increased in the TBI group (*p* < 0.001, Figure 5). Unexpectedly, no differences in the numbers of microglial or astroglial cells between groups were found in the ipsilateral hippocampus. Acute CS elevation positively correlated with hippocampal astrogliosis; the number of astroglial cells in CA3 area of both hemispheres was higher in animals with elevated CS levels on day 3 (*p* < 0.05). We also found that blood CS levels 3 months after TBI negatively correlated with microglial cell density in CA3 areas (*p* < 0.05), presumably mediating the anti-inflammatory effects in survived rats (Figure 5).

## 3. Discussion

In this study, we aimed to evaluate CS-dependent behavioral and morphological changes 3 months after TBI. During the acute post-traumatic period, we showed significant elevations of CS levels on day 3 after TBI, which was accompanied by early CS-dependent objective memory impairment in NORT. Importantly, we revealed that CS elevation on day 3 predicted delayed mortality (CS > 860 nmol/L predicted mortality with an accuracy of 0.947). These data have high translation potential, as in patients with TBI, the glucocorticoid level is also critically important for survival; decreased cortisol levels [18] as well as highly increased levels of both predict delayed mortality in patients [19].

Three months after TBI, we detected delayed mild spatial memory disturbances accompanied by ipsilateral neuronal loss in the DG, microglial cell density increase in the contralateral DG and bilateral moderate thinning of granular and pyramidal cell layers in the hippocampus. A positive correlation of astroglial cell density in the DG with acute CS elevation and a negative correlation of hippocampal area with chronic CS levels in survived rats were found. Unexpectedly, neurodegenerative alterations (neuronal cell loss) and microglial cell density increases were less prominent compared to the data received during the first week after TBI [15,16].

Associations of TBI-induced chronic neuroinflammation and neurodegeneration with cognitive and emotional disorders are actively debated [20]. During the first week after brain trauma, early development of astrogliosis in the neocortex was shown [7,8,9]. Previously, we reported that bilateral neuronal death and glial activation were detected in the DG and CA3 fields of the hippocampus [21]. Similar changes in the contralateral hippocampus, though less pronounced, were also detected in other studies [10,11,12]. GABAergic neurons (parvalbumin, calretinin, somatostatin and neuropeptide Y-immunoreactive) in the polymorphic layer of the DG are among the most vulnerable neuronal populations.

Numerous studies showed cognitive deficits in rat and mice TBI models in parallel with morphological changes, including acute posttraumatic changes and delayed cognitive impairments. Transient impairments of performance in fear conditioning [13] and learning and memory deficits in the Morris water maze [6,14] were reported. A recent study showed remote sex-specific deficits in anterograde but not retrograde memory after LFPI; specifically, male TBI mice were deficient in transferring information from one day to the next in the Barnes maze test during learning, reflecting a retrieval deficiency [22]. In another study, it was shown that rats after LFPI were less likely to develop a spatial strategy during navigation in the Barnes maze [23]. Short-term memory impairments in NORT were also demonstrated 3 and even 6 months after TBI [24].Overall, specific behavioral deficits depended on the test used, as well as on specific features of rodents and experimental designs, but as a rule, they were rather moderate.

The hypothalamic–pituitary–adrenal axis is a key mediator of stress–immune pathway communication following TBI [25]. Suppression of inflammation is among the well-established systemic effects of glucocorticoids (GCs) [26], but, depending on the degree and duration of exposure, GCs in the brain may act as pro-inflammatory agents as well [27,28,29]. GCs in the rat hippocampus or blood may worsen LPS-induced neuroinflammation [30] and enhance the inflammatory response [31,32]. Diffuse TBI also primes microglial cells and promotes depressive-like behavior after the secondary LPS injection [33]. This is not surprising because GCs regulate neuronal excitability, neuroinflammation, increase the vulnerability of neurons to excitotoxicity and may play a key role in hippocampal sensitivity to initial excitotoxic damage [34,35]. TBI also modulates stress reactivity: a blunted CS response to restraint stress after TBI was reported earlier [36], and changed stress-reactivity after TBI was confirmed by the results of the present study (CS response to brief forced swim stress).

**Figure 6:**
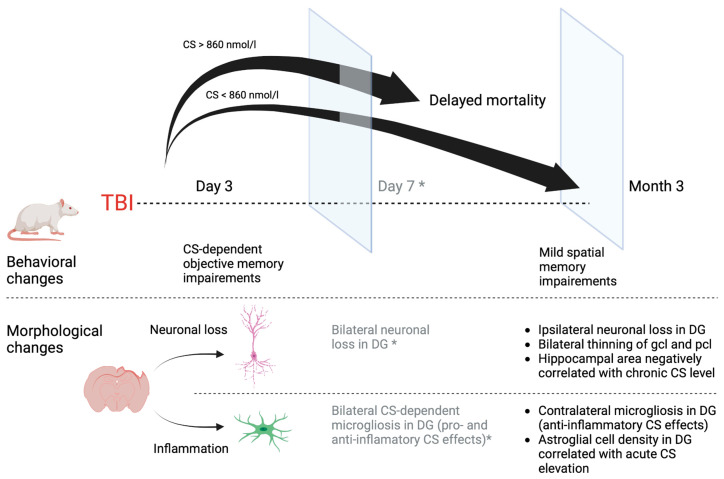
Behavioral and morphological changes in early and late posttraumatic periods. Neuronal cell loss only in the ipsilateral hippocampus accompanied by mild behavioral deficits was revealed in rats 3 months after TBI. In contrast to the early posttraumatic period, these results may be associated with CS-dependent survivorship bias. *—according to our previously published data [15,16], CS—corticosterone, DG—dentate gyrus, gcl—granular cells layer, pcl—pyramidal cells layer.

As mentioned above, according to our previous results [15,16], the delayed neurodegeneration revealed in this study was qualitatively different compared to acute neurodegeneration; bilateral neuronal loss and neuroinflammation took place in the hippocampus 7 days after TBI versus unilateral neuronal loss 3 months after trauma in this study (Figure 6). Obviously, we could expect neurodegeneration in the hippocampus during the remote period, or at least no sooner than during the first week after TBI. One of the most likely explanations of this contradiction may be obvious survivorship bias.

Survivorship bias (or survival bias) is a logical error that is a form of selection bias: concentrating on things that passed a selection process while overlooking those that did not. Survivorship bias is a cognitive shortcut that makes researchers ignore everything that did not survive a specific selection process, focusing instead on only the “winners” and leading to wrong conclusions because of incomplete data [37]. Survivorship bias, a common form of research bias, can lead to poor decision-making in different areas, including finance and business, and it gained lot of attention in these areas. Survivorship bias is important in many medical fields [38,39,40,41,42,43,44], but it is extremely rarely discussed in experimental biomedical research [45].

According to our hypothesis on remote (secondary) glucocorticoid-induced neuroinflammation-mediated hippocampal damage as a result of focal neocortical damage [2,17,29], we expected an increase in neurodegenerative hippocampal changes and related behavioral deficits during the remote period. However, in our study, the remote behavioral disturbances were quite moderate and were revealed only in several behavioral tests. Histological studies revealed moderate neuroinflammatory and neurodegenerative alterations mostly in the ipsilateral hippocampus, which were less expressed than those described during the first week after TBI when these processes were obvious in the DG bilaterally [15,16].

It is noteworthy that CS elevation is regarded as an essential trigger of hippocampal neuroinflammation-mediated neurodegeneration, and its occurrence is a strong predictive index of animal mortality. This may mean that the animals with the highest levels of acute CS secretion that are thus potentially at risk of severe hippocampal damage and gross behavioral disturbances did not survive to the last remote point of the study, thereby preventing us from seeing the whole picture. If we interpreted the results of this study without taking into account the TBI-induced, CS-dependent mortality of rats, then we would have made a logical mistake, as sampling bias would have occurred, as only animals with moderate but not severe posttraumatic CS elevation survived. In other words, the expected severe and bilateral CS-induced neurodegeneration in the hippocampus and behavioral deficits might be masked by survivorship bias after TBI. Is there a way to overcome survivorship bias in such an experimental design? Unfortunately, we do not see better designs because TBI is accompanied by high mortality (both acute and remote) in both human patients and experimental animals when ample TBI models are used. Thus, we just have to understand the limitations of interpreting the data received using severe brain damage models in rodents.

## 4. Materials and Methods

### 4.1. Animals

The experiment was performed on 51 adult (6 months old, approx. 340 g BW) male Sprague–Dawley rats allocated to 3 groups: control (*n* = 9), Sham (*n* = 16) and TBI (*n* = 26). The animals were housed with spruce shavings, provided with bedding and food and water access ad libitum. All experiments with animals were performed in accordance with the EU Directive 2010/63/EU. The Ethical Commission of the Institute of Higher Nervous Activity & Neurophysiology RAS approved our protocol (protocol # 2, 24.05.2017). All efforts were made to minimize the animals’ suffering.

### 4.2. Lateral Fluid Percussion Injury Modelling

We used lateral fluid-percussion injury (LFPI) in rats as a model of TBI. This model is well-described and was chosen because it highly precisely reproduces the TBI conditions seen in human patients. All experimental animals in TBI and Sham operation groups were subjected to total inhalational anesthesia using 2% isoflurane. After skull exposure through a midline incision, a 3 mm wide trepanation aperture was created in the right parietal bone (AP = 3 mm, L = 3 mm) right above the sensorimotor cortex. A plastic head from a Luer-type needle was attached to the aperture with acrylic glue to serve as a hub for applied pressure from the LFPI device (Fluid Percussion Device with the PC-Based Pressure Measurement Unit, Model FP302, Richmond, VA, USA). Once the animals regained full consciousness after the operation, they were placed in a square Styrofoam box measuring 60 cm on each side with walls 40 cm high to maintain the animal and protect it from additional trauma during a seizure. An LFPI (2.5 ± 0.03 atm) was delivered through the aperture. Sham rats were subjected to all surgical procedures (narcosis, craniotomy and connection to LFPI device) except LFPI delivery. After all recordings were completed, the animals were returned to their home cages. Experimental animals were euthanized 3 months later, 30 min after a brief 5-min forced swim test to assess stress reactivity. The animals were euthanized under general chloralhydrate anesthesia through arterial perfusion with a 4% solution of formaldehyde. Before perfusion, we sampled blood through the tail vein. After perfusion, the brains were fixed in the same solution.

### 4.3. Behavior Testing

For behavior assessment, we used the open field test (OFT), elevated plus maze (EPM), object location test (OLT), new object recognition test (NORT) and Barnes maze. Test sessions were performed in the acute period of TBI (days 3–8 after craniotomy) and 3 months after TBI. All test sessions were recorded with a video camera and were then analyzed using the Noldus EthoVision XT.

The OFT was used to evaluate anxiety in rats. We tested animals on day 3 after trauma and 3 months later. The test is based on opposite tendencies in behavior of rodents: their preference of shelter to open space and, on the other hand, a drive to explore their surroundings. The ratio of time spent in the open field to time spent in the shelter indicates the level of anxiety of the experimental animal. The OFT was performed in a round arena bordered by high vertical barriers. The arena was divided into three zones in a manner resembling an archery target: a central zone, an inner circular zone and a zone containing all peripheral regions. The rat was placed in the center of the arena. We recorded the time spent by the animal in each region; frequency and sequence of movements between regions; movement speed and total distance traveled; general level of activity; frequency of exploratory behavior (e.g., using hind legs to stand vertically) in the central and peripheral regions, measuring leaning onto the arena border separately; frequency of defecation and urination. The OFT lasted for 5 min.

The elevated plus maze (EPM) test, used for testing anxiety, took place in a cross-shaped arena with two uncovered arms, two closed arms and high borders providing shade [46]. We tested animals on day 3 after trauma and 3 months later. At the start of the session, we put the rat in the center of the arena. We recorded the latency while leaving the central zone; duration of being in light and dark zones; mean velocity; frequency of transition between zones and exploratory behavior (e.g., head dipping over the side of the arena and standing vertically). A low frequency of exploratory behavior as well as a long latency of leaving the central zone is indicative of anxiety-like behavior. The duration of each session was 5 min.

The object location test (OLT) and new object recognition test (NORT) are designed to evaluate spatial memory and object recognition memory (as in [47] with modifications). The tests lasted for 2 days and were performed during the acute period of TBI (days 4–5 days after TBI) and the remote period (3 month after TBI). On the first day, a habitation session was conducted: rats were put in the arena without any objects and could freely investigate it for 10 min. We recorded the mean velocity and distance moved; these indices were used to assess the motor activity of the animal. On the second day, 3 trials were performed: the training session (the rat was put into the arena with 2 identical objects), the OLT session (one of the objects was moved to the opposite corner of the arena), and the NORT session (the object that was stationary in the OLT was replaced with a novel object). The duration of each session was 5 min with an inter-trial time of 20 min. We recorded values representing the activity of the animal’s investigation of the environment: the frequency of approaching each object and the duration of sniffing. We also recorded mean velocity, total distance traveled and the frequency of zone transitions. The discrimination index was calculated using the following formula: (tA − tB)/(tA + tB), where tA and tB are the sniffing durations of objects A and B, respectively, in seconds.

The Barnes maze was designed to assess spatial learning and long-term spatial memory in rodents (Ref. [48] with modifications). It is based on the ability of rats to orient themselves in space according to visual cues and use their instincts to research the environment and look for shelter. The test setup consisted of an elevated circular platform with 40 evenly spaced black-colored holes around the perimeter. Only one of them represented the true tunnel that the rat could enter to escape the arena, and its location was fixed. Four distal cues surrounded the arena: a triangle, a square, a cross and a circle. At the start of the test session, the experimenter put the animal in the center of the arena. We recorded the time taken to locate and enter the escape tunnel and the type of search strategy employed. The enter delay was calculated as “latency to enter − latency to locate”. During training, rodents typically used a sequence of three different search strategies: random (chaotic checking of holes), serial (serial checking hole-by-hole clockwise or counterclockwise) and spatial (moving directly to the escape tunnel). The learning session set began 7 days before craniotomy. It included one session on day 1, two sessions daily on days 2–6, and one control session on day 7. The test session was conducted on days 6–8 after craniotomy (1 session per day). Three months after craniotomy, we first tested animals with the same escape tunnel position as during previous sessions; then, the escape tunnel was rotated 180 degrees, and tests based on learning its new position were performed for the next 6 consecutive days. The duration of every session was 5 min.

### 4.4. Histology, Immunohistochemistry and Morphometry

Vibratome sections of 50 μm thickness were prepared from fixed rat brains. Sections located 600 μm apart with coordinates between 2.1 and 5.8 mm from the bregma were selected for analysis. Sections were stained using the Nissl method with Cresyl Violet dye. For immunohistochemical staining (ionized calcium-binding adaptor molecule 1, Iba 1, a microglial marker), floating slices were washed in PBS and then rinsed in PBST (0.01-M PBS with 0.3% Triton X-100). The slices were incubated in a blocking solution (5% normal goat serum (MP Biomedicals, Irvine, CA, USA) in PBST and then incubated overnight in a solution containing rabbit anti-Iba1 antibody (Wako, Neuss, Germany) or in a solution containing rabbit polyclonal antibody to GFAP (1:500, DAKO, Glostrup, Denmark) at 4 °C. Then, the sections were thoroughly washed in PBST and incubated in a solution containing Anti-Rabbit-IgG conjugated with a peroxidase antibody produced in goats (1:500, Sigma-Aldrich, Darmstadt, Germany) for 2 h. Slices were stained using a fast DAB with metal enhancer (Sigma-Aldrich, Darmstadt, Germany), mounted and coverslipped.

Microphotographs of sections stained by using the Nissl method were made using a Keyence BZ-X700 microscope (Itasca, IL, USA) for light microscopy. First, four sections with magnification 20 were selected to count the total number of neurons in the polymorphic layer of DG and the CA1 and CA3 areas of the hippocampus in both hemispheres. Hence, area on slices of the above-mentioned areas and total hippocampal areas were measured with magnification ×2. Microphotographs of immunohistochemically stained sections were made using a ZEISS (Oberkochen, Germany) for apotome microscopy (magnification ×20). The density of microglial, astroglial and neuronal cells was calculated as the number of cells in a 150 × 150 μm visual field in the polymorph layers of the DG, CA1 and CA3 areas of the hippocampus. Further calculations of microglial cells were made using the ImageJ 1.52q program.

### 4.5. Assessment of CS

Tail blood plasma was collected before all behavioral tests, on day 3 and 7, and 1, 2 and 3 months after craniotomy. Aliquots were stored at −80 °C. Enzyme-linked immunosorbent assay kits were used for measurements of corticosterone (Corticosterone ELISA, DRG, Marburg, Germany) according to the manufacturer’s instructions.

### 4.6. Statistical Methods

Statistical analysis was performed using Jamovi ver. 2.3.21. Visualizations were made using Graph Prism 8 and BioRender.com (Figure 6). The normality of distribution was assessed using the Shapiro–Wilk test. Three groups (control, Sham and TBI) were compared using the Kruskal–Wallis test with post-hoc analysis (Dwass–Steel–Critchlow–Fligner test). The Mann–Whitney U test was used to compare independent variables (CS in surviving animals vs. deceased animals). The Wilcoxon test was used for dependent variables (CS levels before and after forced swim test). The accuracy of prediction was assessed using ROC analysis. Survival rates were compared with the Kaplan–Meier method. For continuous variables, Spearman’s correlations were calculated. All data are presented as Mean ± SEM (standard error of mean).

## 5. Conclusions

Initially, the aim of this study was to evaluate CS-dependent behavioral and morphological changes 3 months after TBI in Sprague-Dawley rats. During the acute post-traumatic period, the elevation of CS levels on day 3 after TBI was accompanied by early CS-dependent objective memory impairments and predicted delayed mortality. Three months after TBI, only mild spatial memory disturbances were revealed, and they were accompanied by ipsilateral neuronal loss in the DG, microgliosis in the contralateral DG and bilateral thinning of hippocampal cell layers. We found a positive correlation of astroglial cell density in the DG with acute CS elevation and a negative correlation of hippocampal area with chronic CS levels in surviving rats. Because the acute CS level was shown to predict mortality and only animals with moderate but not severe posttraumatic CS elevation survived, late CS-induced morphological and behavioral deficits may be masked and underestimated due to CS-dependent survivorship bias.

### Limitations

1. We did not use tissue samples collected from spontaneously dead animals to confirm bilateral hippocampal damage in rats with CS elevation > CS > 860 nmol/L because of the impracticability of standardizing the delay of tissue collection to avoid unbiased assessment.
2. We used the same cohort of animals with the same behavioral tests in the acute period of TBI and 3 months after. The lack of possible differences in the second trial of cognitive tests could be possibly explained by experience bias. To avoid bias, we used three groups of animals (control, Sham and TBI), long inter-trial periods for repetitive testing (3 months) and the re-learning paradigm in the Barnes maze test.
3. We used a single immunohistochemical marker for each type of glial cell (Iba1 for microglial cells and GFAP for astroglial cells). It was not enough to prove cell types in all visible marker-positive cells, and additional makers might improve the accuracy of the assessment. Immunohistochemistry also cannot be used to assess changes in gene and protein expression in cells without precise immunochemical analysis (Western blot).

## Data Availability

Not applicable.

## Supplementary Materials

The supporting information can be downloaded at: https://www.mdpi.com/article/10.3390/ijms24054542/s1.

## Abbreviations

CS	corticosterone
DG	dentate gyrus, hippocampal field
EPM	elevated plus mase
gcl	granular cells layer
GCs	glucocorticoids
HPA	hypothalamo-pituitary axis
LPS	lipopolysaccharide
LFPI	lateral fluid percussion brain injury
NORT	new object recognition test
OF	open field test
OLT	object location test
pcl	pyramidal cells layer
TBI	traumatic brain injury
