# Supplementary material for: Delayed TBI-Induced Neuronal Death in the Ipsilateral Hippocampus and Behavioral Deficits in Rats: Influence of Corticosterone-Dependent Survivorship Bias?
Source: Int J Mol Sci. 2023 Feb 25;24(5):4542. doi: 10.3390/ijms24054542 (PMC10003069; doi:10.3390/ijms24054542)
Supplement: Supplementary file 1 [file ijms-24-04542-s001.zip › ijms-2164168-supplementary.pdf]

International Journal of
Molecular Sciences

MDPI

Supplementary materials to the manuscript "Delayed TBI-induced neuronal death in the ipsilateral hippocampus and behavioral deficits in rats: Influence of corticosterone-dependent survivorship bias?" by Ilia Komoltsev, Daria Shalneva, Olga Kostyunina, Aleksandra Volkova, Stepan Frankevich, Natalia Shirobokova, Anastasia Belikova, Sofia Balan, Olesya Chizhova, Olga Salyp, Daria Bashkatova, Pavel Kostrukov, Aleksandra Solovyova, Margarita Novikova and Natalia Gulyaeva

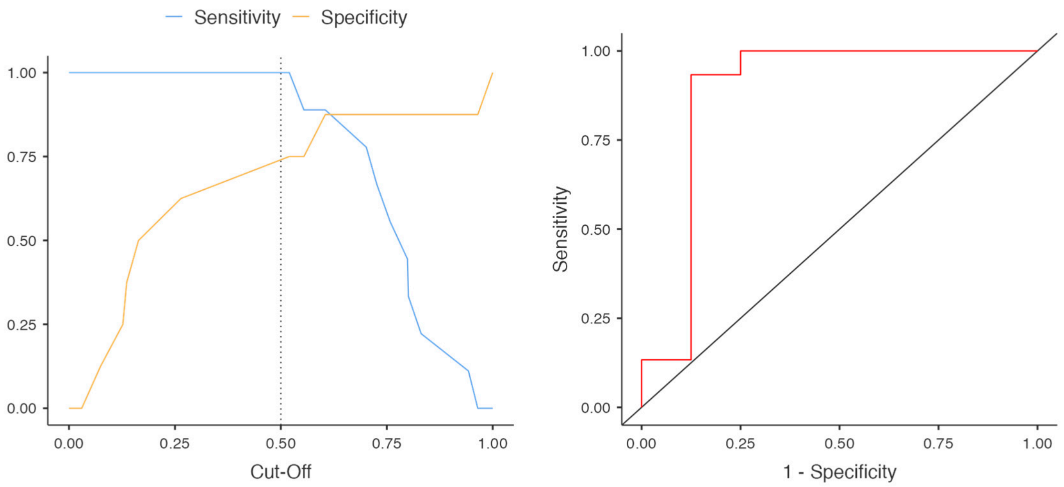

Figure S1. Cut-off plot and ROC-curve for the ROC analysis of CS level on day 3 as the mortality predictor.

Table S1. Predictive measures of ROC-analysis of CS level on day 3 as the mortality predictor.

Predictive Measures			
**Accuracy**	**Specificity**	**Sensitivity**	**AUC**
0.947	0.750	1.00	0.883

Note. The cut-off value is set to 0.7

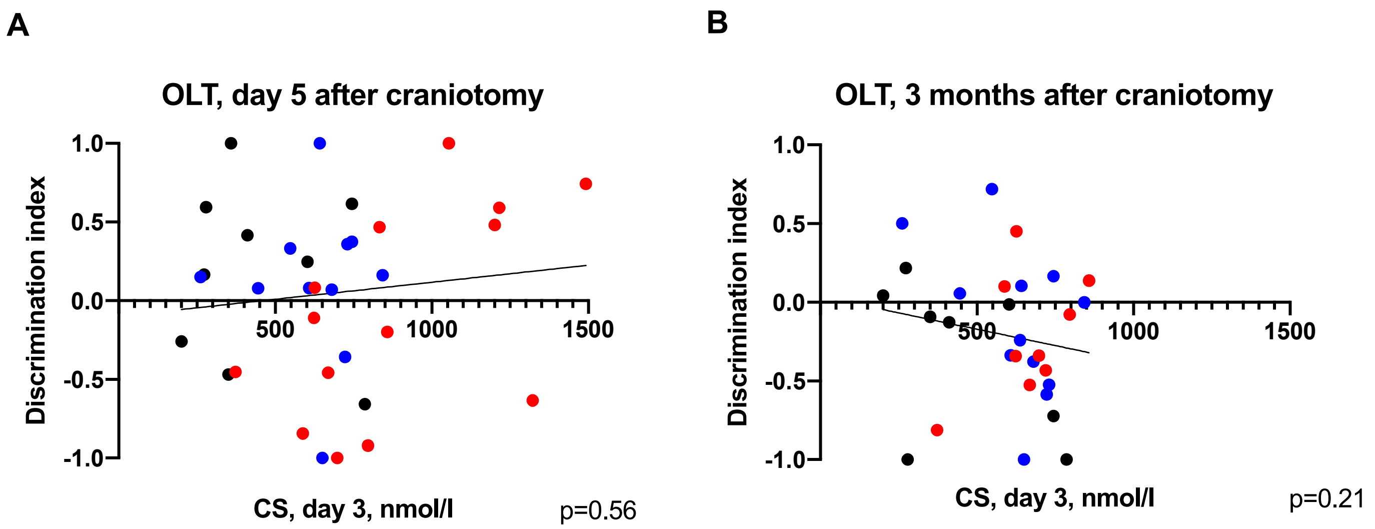

Figure S2. Correlation of CS on day 3 after TBI with discrimination index in OLT on day 5 after craniotomy and 3 months later.

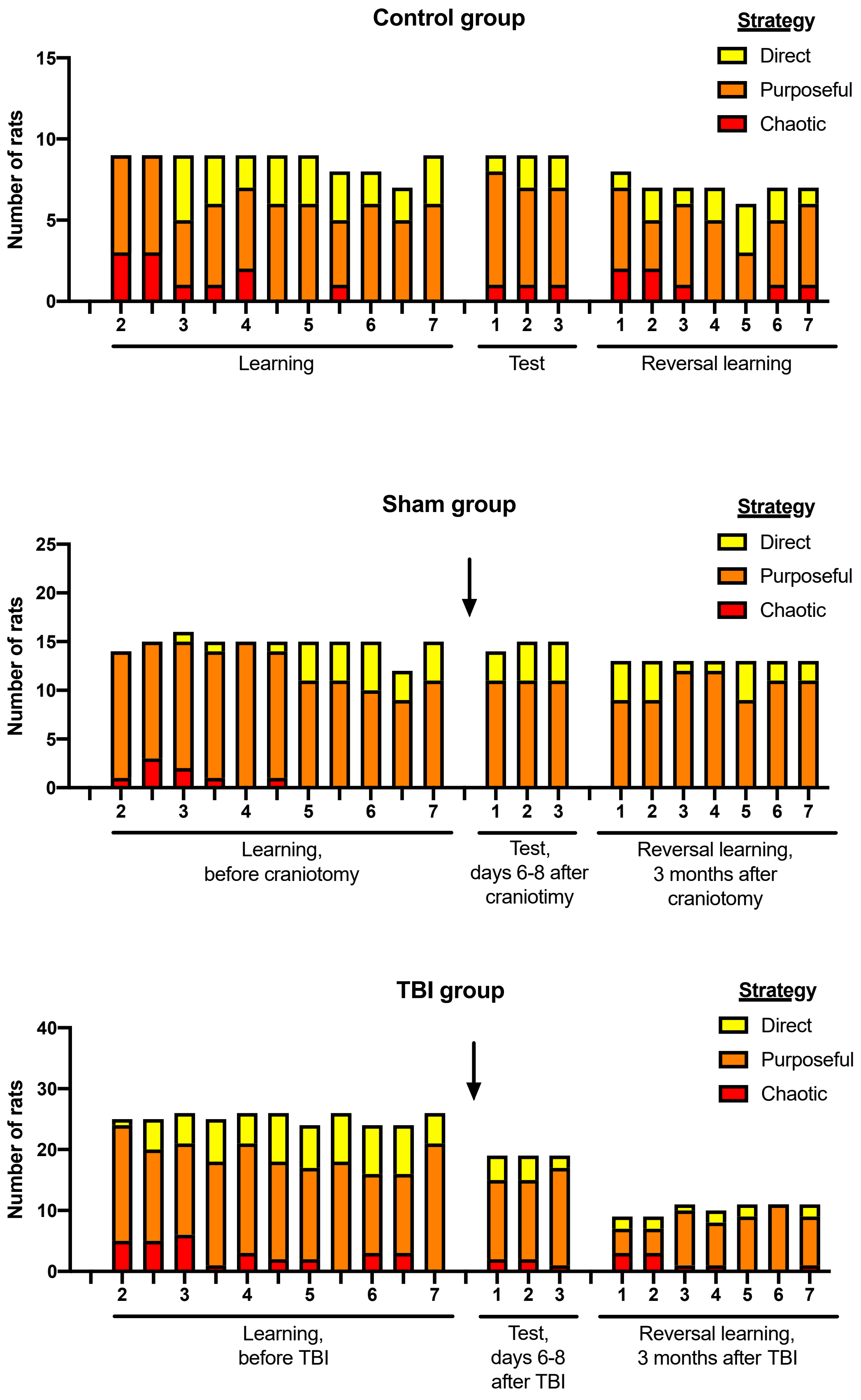

Figure S3. Different strategies in Barnes maze test.

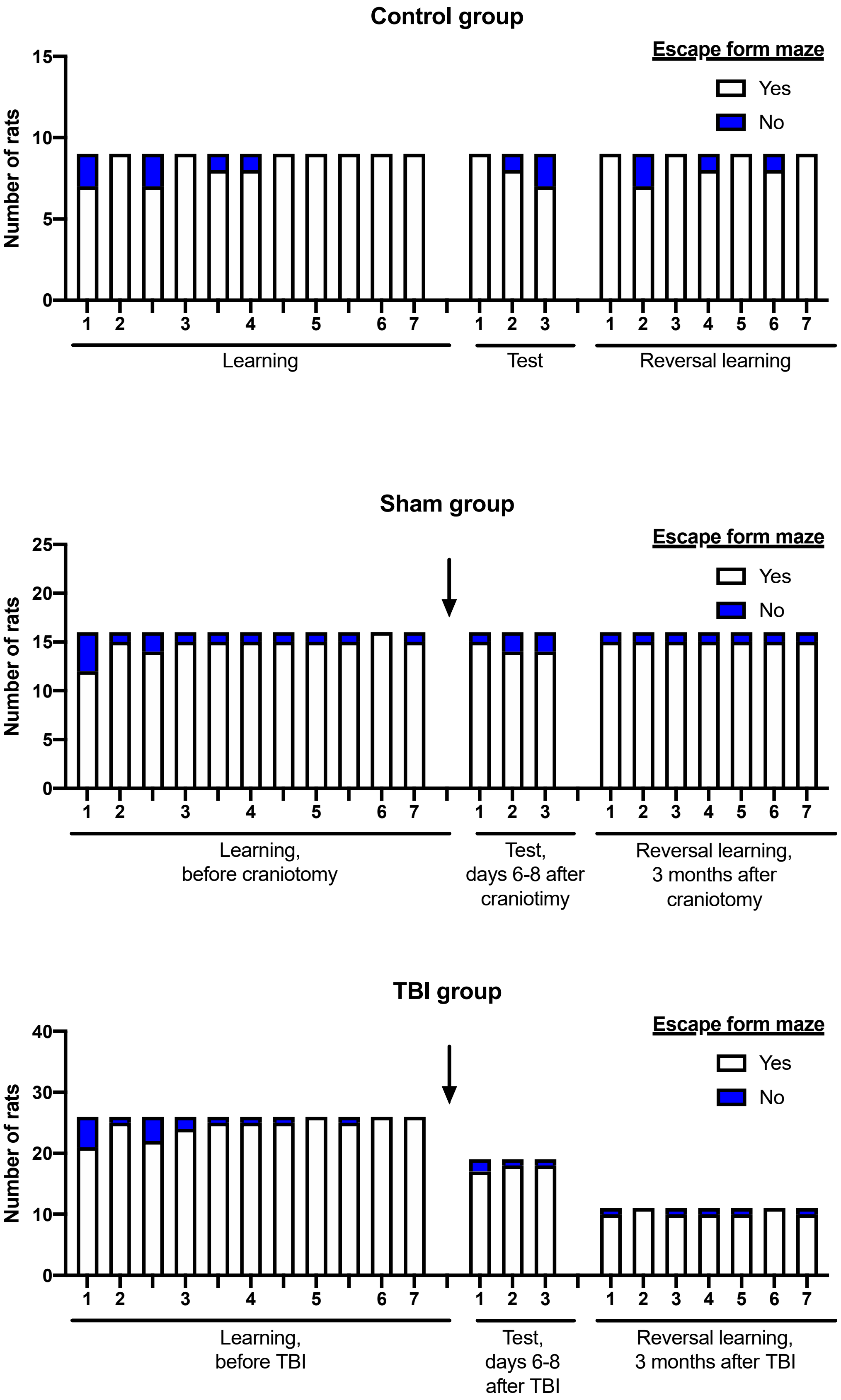

Figure S4. Escapes out of Barnes maze proportions.

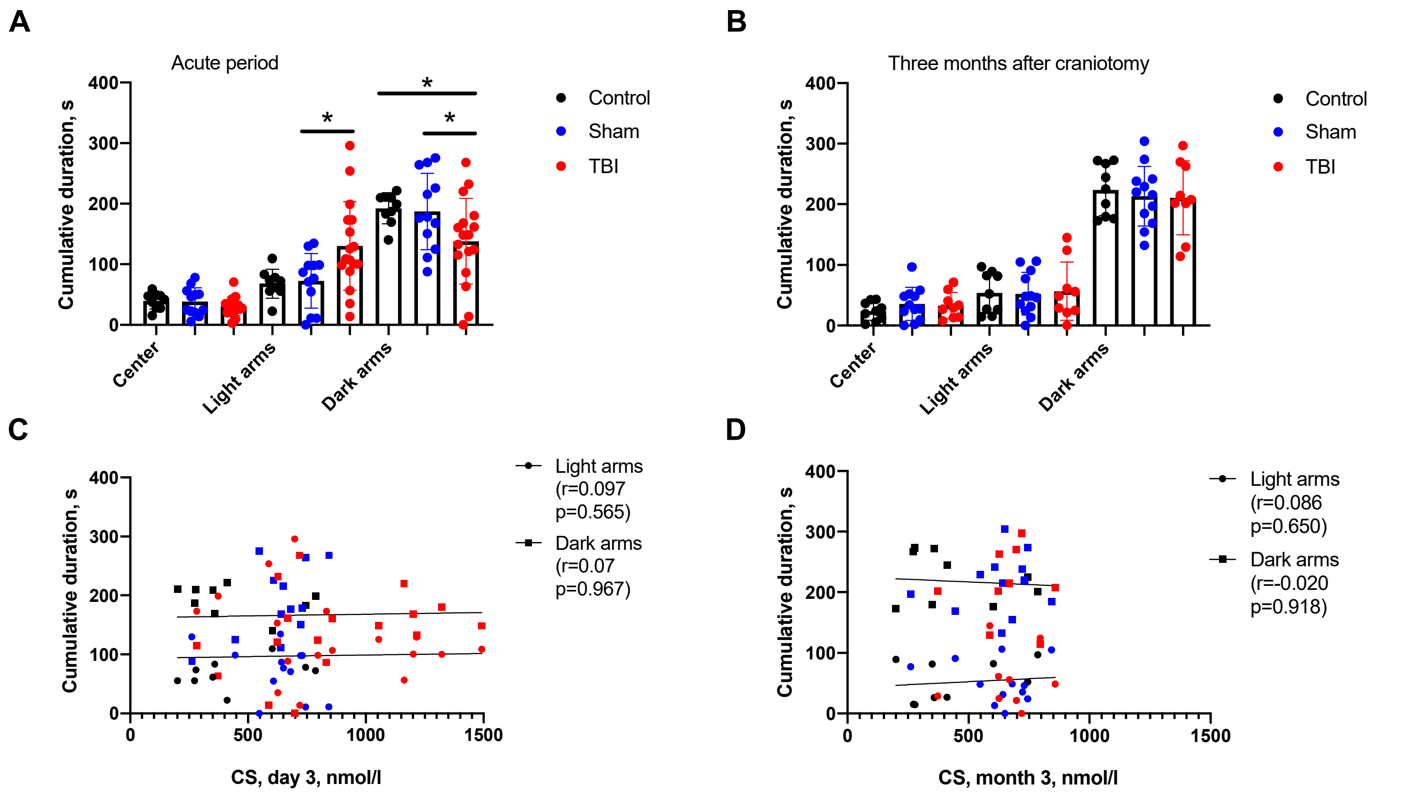

Figure S5. Behavioral changes in elevated plus maze. A, B – Cumulative duration in the arms in acute and remote TBI periods. C, D - correlation of CS on day 3 after TBI or month 3 after TBI with cumulative duration in the arms of EPM. * - Kruskal-Wallis test with post-hoc analysis.
